# Sex differences in depression in Parkinson’s disease: cognitive dysfunction and female-specific associations

**DOI:** 10.3389/fnins.2025.1704461

**Published:** 2025-12-16

**Authors:** Huashuo Zhao, Yi Sun, Qiushuang Wang, Zixuan Zhao

**Affiliations:** 1 School of Public Health, Xuzhou Medical University, Xuzhou, Jiangsu, China; 2 School of Public Health, Zhengzhou University, Zhengzhou, Henan, China; 3 School of Health Economics and Management, Nanjing University of Chinese Medicine, Nanjing, Jiangsu, China

**Keywords:** cognitions, depression, linear mixed model, Parkinson’s disease, sex differences

## Abstract

**Objective:**

This study used a linear mixed model to explore the relationship between cognitive function and depression in Parkinson’s disease (PD) patients.

**Participants:**

The study data were collected from 450 Parkinson’s disease patients who participated in the Parkinson’s Disease Progress Marker Project (PPMI) from 2010 to 2024, including 176 women and 274 men.

**Measurements:**

Cognitive function was assessed using the Montreal Cognitive Assessment Scale (Moca), and depression was measured using the Geriatric Depression Scale (GDS). The correlation between cognitive function and depression was determined using a linear mixed model.

**Results:**

Anxiety, daily living ability, and autonomic nervous system function are significant factors affecting both men and women. Women experience a stronger impact of anxiety, daily activity limitations, and autonomic nervous system dysfunction on depression. The longer the duration of the illness, the more severe the depression in women. Moreover, cognitive abilities protect against depression only in women. Women exhibit higher individual heterogeneity in baseline depression and greater variability in the rate of change over time, with those who are more depressed showing a more gradual change.

**Conclusion:**

In female Parkinson’s disease patients, there is a negative correlation between cognitive ability and depression, whereas this correlation is not observed in male patients. This study provides new evidence that sex differences influence the relationship between cognitive ability and depression in Parkinson’s disease patients. Future research should consider the role of sex differences in the context of cognitive ability and depression.

## 1 Introduction

Parkinson’s disease (PD) is a common neurodegenerative disorder characterized by motor dysfunction and often accompanied by non-motor symptoms such as depression, anxiety, and cognitive decline (Zhu et al., 2021). As the population ages, the impact of PD on patients’ quality of life and societal burden has become increasingly significant (Schneider and Kortagere, 2022). Depression is a common non-motor symptom in PD patients, significantly affecting their quality of life, disease progression, and prognosis (Kenangil et al., 2023). Numerous studies have shown that the depressive symptoms in PD patients are not solely driven by the pathological factors of neurodegeneration but are also intricately linked to anxiety, cognitive decline, and autonomic dysfunction (Wang et al., 2022). Anxiety can lead to prolonged psychological stress, increasing the risk of depression; meanwhile, cognitive decline can weaken people’ ability to cope with emotional distress, further exacerbating depressive symptoms (Sun et al., 2024; Ma et al., 2025). Additionally, motor dysfunction and autonomic dysregulation in PD patients can indirectly worsen depression by affecting daily living independence and circadian rhythms (Kwon et al., 2022; Klimanova et al., 2024). However, few studies have explored the sex differences in these factors during the development of depression, and sex may play a crucial role in regulating the relationship between emotions, cognition, and neurodegeneration. The aim of this study was to analyze the effects of autonomic dysfunction and cognitive function on the degree of depression in Parkinson’s disease patients. A linear mixed effects model was constructed to examine the heterogeneity of these relationships in men and women, to provide a basis for more accurate clinical intervention strategies.

## 2 Materials and methods

### 2.1 Data sources

We obtained data from the PPMI database and included 450 patients with Parkinson’s disease who were registered between 2010 and 2024. Data used in the preparation of this article were obtained on [2024-11-25] from the Parkinson’s Progression Markers Initiative (PPMI) database: a large, longitudinal, international observational study aimed at identifying biomarkers of disease progression in newly diagnosed, untreated Parkinson’s disease patients,^1^ RRID: SCR_006431. For up-to-date information on the study, visit http://www.ppmi-info.org.

To construct a longitudinal cohort with complete core assessment trajectories, we applied two sequential inclusion criteria: Baseline data completeness: Participants missing essential baseline demographic or clinical information—including sex, age, and race—were excluded (*n* = 15). Follow-up completeness: Only participants with continuous annual assessments from baseline through Year 4 (i.e., five visits in total) were retained for analysis.

**Figure 1:**
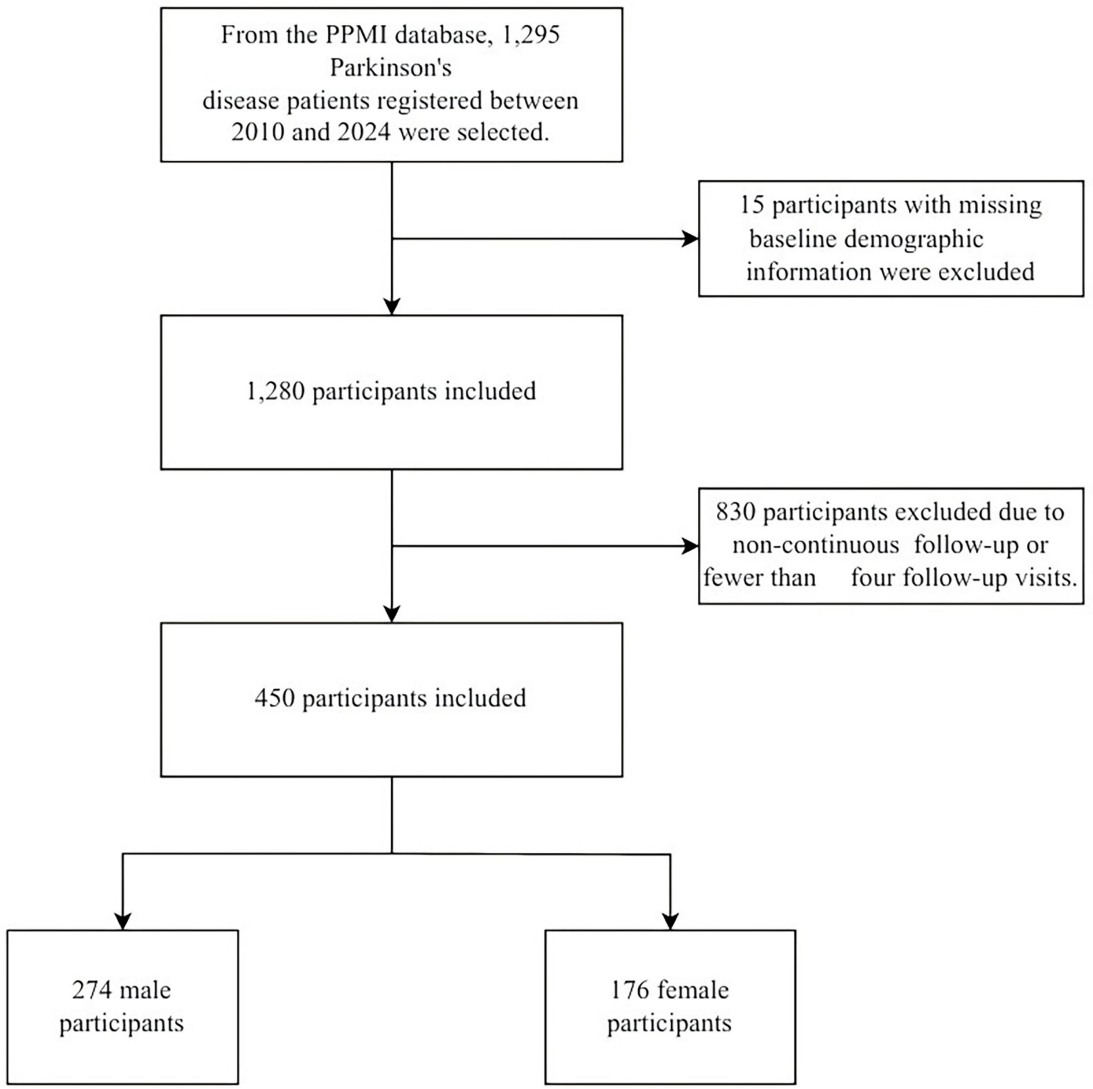
Participant inclusion flowchart.

Following this screening process, the final analytic sample comprised 450 participants, as illustrated in Figure 1.

After this screening, sporadic missing values remained in clinical scales (e.g., GDS items, Moca scores). To preserve statistical power and mitigate selection bias, we implemented Multiple Imputation by Chained Equations (MICE) using R mice (v3.17.0). Key specifications: Variables in imputation: All analytic variables – including longitudinal GDS (response), cognitive scores (Moca), time, sex, age, MDS-UPDRS II/III, STAI, and subject ID for random effects linkage. Imputation models: Predictive mean matching (pmm) for continuous variables (e.g., GDS), logistic regression (logreg) for binary outcomes.

Output: Generated m = 50 complete datasets after 10 iterations (convergence verified via trace plots).

Analysis integration: Linear mixed models were fitted to each imputed dataset individually, with pooled estimates derived via Rubin’s rules.

To evaluate potential attrition bias, we compared baseline characteristics between participants retained in the analytic sample (*n* = 450) and those excluded due to incomplete follow-up (*n* = 15). No statistically significant differences were observed in baseline GDS scores, MoCA total scores, MDS-UPDRS Part III scores, or age, suggesting minimal systematic dropout bias. Nevertheless, the influence of unmeasured confounders (e.g., socioeconomic status or access to care) cannot be ruled out.

### 2.2 Clinical evaluation and scales

The Geriatric Depression Scale (GDS) (Yesavage et al., 1982; Smarr and Keefer, 2011; Bossola et al., 2023) is a specialized tool designed to assess the severity of depressive symptoms in older adults. Developed by Yesavage et al. (1982), it aims to address the limitations of other depression assessment tools when applied to older populations. The State-Trait Anxiety Inventory (STAI) (Knowles and Olatunji, 2020) is a widely used psychological assessment tool for measuring an individual’s anxiety levels. The Movement Disorder Society–sponsored revision of the Unified Parkinson’s Disease Rating Scale (MDS-UPDRS) is an updated version of the original UPDRS, developed by the International Parkinson and Movement Disorder Society to provide a more precise and comprehensive assessment of Parkinson’s disease symptoms and disability (Alam et al., 2025). MDS-UPDRS Part II evaluates the impact of Parkinson’s disease on patients’ activities of daily living (ADL). MDS-UPDRS Part III assesses motor function, typically during the medication “on” state—when symptoms are best controlled—but can also be used to evaluate motor severity during the “off” state, when medication effects wear off or are absent. The MoCA scale (Montreal Cognitive Assessment) is a rapid screening tool for mild cognitive impairment, encompassing various cognitive domains such as attention, executive function, memory, language, visuospatial ability, abstract thinking, calculation, and orientation, with a total score of 30 points (Islam et al., 2023). SCOPA-AUT (Scales for Outcomes in Parkinson’s Disease – Autonomic) is a scale specifically used to assess autonomic dysfunction in patients with Parkinson’s disease (Westergren et al., 2022). Among them, the Urinary Sub-score is a subscale of this scale, used to evaluate autonomic symptoms related to the urinary system. Sdmtotal (Symbol Digit Modalities Score) is a key clinical measure of an individual’s information processing speed and overall cognitive efficiency (Thomason et al., 2025). H&Y, The Hoehn and Yahr scale is used to assess the severity of Parkinson’s disease, ranging from stage 1 to stage 5, reflecting disease progression from mild unilateral symptoms to complete bedridden status (Kahn et al., 2019).

### 2.3 Statistical analysis

We employed sex-stratified Least Absolute Shrinkage and Selection Operator (LASSO) regression to identify robust predictors of depression severity while mitigating multicollinearity and overfitting risks. Implementation utilized the glmnet package (R v4.1.3) with *λ* optimization conducted via 10-fold cross-validation. Model selection primarily adhered to the λ.1se criterion (the largest λ value within one standard error of the minimum root mean squared error). Performance was quantified through the coefficient of determination (*R*^2^), mean squared error (MSE), and its square root (RMSE). Separate tuning grids were applied for male and female cohorts.

Then, baseline characteristics were compared between female and male participants. Continuous variables with normal distribution were analyzed using independent-samples *t*-tests, while non-normally distributed continuous variables and ordinal categorical variables were compared using the Mann–Whitney U test. Categorical variables with nominal scales were assessed using chi-square tests. Disease duration and education level were z-score standardized to improve model convergence and interpretability. Trait anxiety, as measured by the State-Trait Anxiety Inventory (STAI), was decomposed into between-person (group-mean centered) and within-person (person-mean deviation) components following the recommended approach for longitudinal analysis of time-varying covariates.

Separate LMMs were fitted for male and female participants to account for potential sex-specific associations. Each model incorporated a random intercept to capture individual heterogeneity in baseline depression levels and a random slope for follow-up time to accommodate inter-individual differences in the rate of depressive symptom change. Model parameters were estimated using maximum likelihood (ML), providing a balanced framework for estimating both fixed and random effects. Model fit was evaluated and compared using information criteria: the female model demonstrated superior fit (AIC = 3604.5, BIC = 3690.6) relative to the male model (AIC = 5464.4, BIC = 5558.4). Diagnostic checks—including residual-versus-fitted plots and Q–Q plots—confirmed that the assumptions of residual normality and homoscedasticity were adequately met.

## 3 Results

### 3.1 Baseline information of participants

A total of 450 patients with Parkinson’s disease were included, of whom 176 were women (39.1%) and 274 were men (60.9%). Baseline information for each variable is reported in Table 1.

**Table 1:** Baseline information.

Variable	Altogether	Men	Women	*p*
	450	274	176	
Age	61.4 ± 9.7	61.7 ± 9.7	60.1 ± 9.6	0.390
Race				0.349
White	425 (94.0%)	261 (95.3%)	164 (93.2%)	
Non-white	25 (6%)	13 (4.7%)	12 (6.8%)	
Family history				0.960
1st degree family w/PD	102 (22.7%)	63 (23.0%)	39 (22.2%)	
Non-1st degree family w/PD	55 (12.2%)	34 (12.4%)	21 (11.9%)	
No family w/PD	293 (65.1%)	177 (64.6%)	116 (65.9%)	
STAI	66.2 ± 17.8	64.9 ± 17.8	69.3 ± 19.9	0.015
SCOPA-Ur	4.9 ± 3.2	4.6 ± 3.2	4.2 ± 3.1	0.291
EDUCYRS	16.0 ± 3.2	16.0 ± 3.2	14.6 ± 3.8	<0.001
Duration	14.2 ± 14.5	11.4 ± 14.5	18.7 ± 21.8	<0.001
Moca	26.4 ± 2.4	26.8 ± 2.4	26.8 ± 3.0	0.861
UPDRS2_score	8.1 ± 4.5	6.2 ± 4.5	6.0 ± 5.3	0.714
UPDRS3_score_on	21.4 ± 9.1	19.9 ± 11.7	18.3 ± 8.6	0.056
Sdmtotal	39.9 ± 10.0	40.4 ± 10.0	41.9 ± 10.9	0.125
GDS-15	2.8 ± 2.3	2.4 ± 2.3	2.8 ± 3.0	0.114
H&Y				0.111
0	1 (0.2%)	1 (0.4%)	0 (0.0%)	
1	189 (42.0%)	121 (44.2%)	68 (38.6%)	
2	253 (56.2%)	151 (55.0%)	102 (58.0%)	
3	7 (1.6%)	1 (0.4%)	6 (3.4%)	

STAI, State-Trait Anxiety Inventory; SCOPA-Ur, SCOPA-AUT Urinary Subscale; Moca, Montreal Cognitive Assessment; UPDRS2, The Movement Disorders Society-sponsored Revision of the Unified Parkinson’s Disease Rating Scale Part II; UPDRS3_score_on, Movement Disorders Society-sponsored Revision of the Unified Parkinson’s Disease Rating Scale Part III Score (assessed in ON medication state); Sdmtotal, Symbol Digit Modalities Score; GDS-15, 15-item Geriatric Depression Scale; H&Y, Hoehn and Yahr scale.

### 3.2 LASSO regression analysis

Sex-stratified analysis revealed divergent regularization patterns across critical thresholds. Using the *λ*.min criterion (minimum cross-validation error), the female model required stronger regularization (λ.min = 0.128) compared to males (λ.min = 0.055; 132.7% difference). At this threshold, women exhibited superior explained variance (*R*^2^ = 0.692 vs. 0.531 in men) and higher cross-validation MSE (3.548 vs. 3.138). At the preferred clinical criterion λ.1se (λ = 0.470 for both sexes), women maintained significantly enhanced predictive performance (*R*^2^ = 0.643 vs. 0.460) despite prediction MSE elevation (4.111 vs. 3.614).

### 3.3 Linear mixed model analysis

To investigate the impact of a series of variables on depression, a linear mixed effects model was constructed, considering individual heterogeneity. The model captures the individual differences in depression scores during the follow-up period of each patient through random intercepts and time slopes. The fixed effects variables in the model include anxiety (mean_stai and stai_centered, both standardized), urinary system autonomic symptoms (scopa_ur), years of education (educyrs, standardized), cognitive function (moca), non-motor symptom score (updrs2_score), motor symptom response to medication (updrs3_score_on), information processing speed (sdmtotal), and baseline disease duration (baseline duration, standardized). Family history and race were then added as covariates for analysis. Regardless of sex, family history and race showed no significant association with depression.

### 3.4 Model result comparison

Regarding fixed effects, stai (mean_stai and stai_centered, both standardized), updrs2_score (daily living activities), and scopa_ur (autonomic nervous system function) are significant factors affecting both men and women. Compared to men, women show a slightly stronger influence of anxiety, daily activity impairment, and autonomic nervous system dysfunction on depression. In Model 1, only the standardized baseline duration of illness and cognitive ability were significantly associated with depression in women. In Model 2, the standardized baseline duration of illness was marginally significant in women, while cognitive ability was only significant in women. This suggests that the longer the baseline illness duration, the more severe the depression, and the protective effect of cognitive ability against depression is observed only in women, as shown in Table 2.

**Table 2:** Fixed effects.

Variable	Women *β* (*p*)		Men *β* (*p*)	
	Model 1	Model 2	Model 1	Model 2
Mean_stai (standardized)	2.045 (<0.001)	2.047 (<0.001)	1.421 (<0.001)	1.409 (<0.001)
Stai_centered (standardized)	0.080 (<0.001)	0.080 (<0.001)	0.070 (<0.001)	0.070 (<0.001)
SCOPA-Ur	0.083 (0.001)	0.084 (0.001)	0.062 (0.002)	0.063 (0.001)
Updrs2_score	0.068 (<0.001)	0.069 (<0.001)	0.049 (<0.001)	0.050 (<0.001)
Baseline duration (standardized)	0.229 (0.046)	0.203 (0.094)	−0.004 (0.960)	−0.006 (0.936)
Moca	−0.073 (0.009)	−0.074 (0.009)	−0.017 (0.438)	−0.015 (0.492)
EDUCYRS (standardized)	−0.067 (0.574)	−0.075 (0.545)	−0.119 (0.145)	−0.110 (0.179)
SDMTOTAL	−0.001 (0.937)	0.000 (0.995)	−0.008 (0.212)	−0.008 (0.250)
Updrs3_score_on	0.009 (0.147)	0.010 (0.130)	−0.003 (0.600)	−0.003 (0.599)

Model 1: no covariates.

Model 2: race and family history were used as covariates.

In terms of random effects, the individual heterogeneity of baseline depression in women was higher and the rate of change over time was more different. The change of high baseline depression in women was more gradual, and a slight positive correlation between men was seen in Table 3.

**Table 3:** Random effects.

Parameter	Model 1	Model 2	Model 1	Model 2
Intercept variance (individual baseline difference)	2.723 (SD = 1.650)	2.752 (SD = 1.659)	1.045 (SD = 1.022)	1.037 (SD = 1.019)
Time slope variance (difference in rate of change)	0.212 (SD = 0.460)	0.216 (SD = 0.465)	0.037 (SD = 0.193)	0.038 (SD = 0.194)
Intercept-slope correlation	−0.61	−0.62	0.02	0.01

Model 1: no covariates.

Model 2: race and family history were used as covariates.

## 4 Discussion

Female patients may exhibit heightened sensitivity to the physiological and psychological impacts of anxiety and functional impairment, a phenomenon potentially linked to the dual influence of social roles and neurobiological mechanisms. Previous studies have demonstrated correlations between social anxiety in women and their physiological responses (Crowley et al., 2020; Man et al., 2023). Our research reveals that female Parkinson’s disease patients are more susceptible to multiple depression risk factors. While depression is associated with cognitive decline in Parkinson’s disease (PD), emerging evidence suggests that even with depressive symptoms, women may experience slower progression to cognitive impairment compared to men (Iwaki et al., 2021). This disparity could be attributed to PD-specific pathophysiological mechanisms, which should not be ruled out as potential effect modifiers. Our findings regarding the protective role of cognitive function in depression among women should be interpreted considering these potential sex differences. Future longitudinal studies with sex-specific stratification are required to fully elucidate temporal correlations.

The sex-specific protective effect of cognitive function on depressive symptoms in Parkinson’s disease patients—observed exclusively in women—may stem from the interaction between estrogen-mediated neurobiological mechanisms and neurodegenerative pathology associated with Parkinson’s disease (Naufel et al., 2021; Wharton et al., 2012; Dong-Chen et al., 2023). Premenopausal women typically benefit from elevated estrogen levels, which enhance neuroplasticity in emotional regulation networks through structural and neurochemical pathways. Estrogen improves structural integrity by upregulating brain-derived neurotrophic factor (BDNF) synthesis, thereby promoting dendritic branching development in the hippocampus and medial prefrontal cortex (Zhang et al., 2024; Sohrabji and Lewis, 2006; Vilor-Tejedor et al., 2020). Simultaneously, estrogen strengthens key neurotransmitter systems: by amplifying mesolimbic dopamine signaling through estrogen receptor beta (ER*β*) in the ventral tegmental area, it increases tyrosine hydroxylase activity, forming a synergistic “cognitive-emotional buffering mechanism” where preserved cognitive function activates relatively intact dopaminergic neural circuits to suppress depressive symptoms. Additionally, estrogen upregulates serotonin transporter (SERT) expression in the nucleus accumbens, partially counteracting the accelerated serotonin depletion observed in Parkinson’s disease patients (Kalinowski and Bogus-Nowakowska, 2024). These mechanisms collectively enable women with higher cognitive reserves to more effectively mobilize anatomically intact and neurochemically active neural networks for symptom alleviation. In stark contrast, men lacking this hormone-supported neuroprotective mechanism exhibit earlier and more pronounced cognitive-emotional regulation degeneration, manifested as accelerated hippocampal functional decline.

Social roles may be a key factor contributing to sex differences. Women typically shoulder the primary responsibility for family care, and cognitive decline may impair their ability to fulfill these roles, thereby exacerbating depressive symptoms. Conversely, maintaining good cognitive function helps women sustain social and family responsibilities, creating a protective feedback mechanism. In contrast, men’s social roles place less emphasis on cognitive performance, resulting in weaker correlations between cognitive abilities and emotional health.

The observed sex differences in depression progression require consideration of three key factors that may introduce confounding effects and unanalyzed mechanisms. First, while the PPMI database records baseline dopamine medication use, sex-specific variations in left-handed dopamine equivalent daily dose (LEDD) may reflect not only motor control demands but also sex-specific drug metabolism and associated emotional side effects. For instance, women may experience transient anxiety during early postoperative rapid LEDD reduction, which could lead to misdiagnosis as depression and subsequent dosage adjustments, thereby affecting long-term non-dopaminergic symptom scores and treatment strategies (Golfrè Andreasi et al., 2022). Future studies should incorporate time-varying LEDD into covariate analysis. Second, although we controlled for MDS-UPDRS-III scores, non-motor fluctuations (NMF) accelerate more rapidly in women and are more closely associated with depression (Donzuso et al., 2023), suggesting persistent residual confounding factors. Third, in sex-disproportionate comorbidities like fibromyalgia, women may experience heightened neuroinflammation exacerbating left-sided kinker bundle dysfunction, thereby increasing depressive risk—particularly when combined with Parkinson’s disease (Tenti et al., 2025). Collectively, these unmeasured mediators may amplify the assessment of female susceptibility. Additionally, our study did not specifically analyze the relationship between glutamatergic-mediated “therapeutic decline” or “dyskinesia” and depression (Pagonabarraga et al., 2021). While dopaminergic deficiency is the core pathological mechanism of PD depression, these non-dopaminergic pathways (manifesting as specific motor complications) represent a crucial direction for future research on sex differences in PD mood disorders.

Research on random effects models reveals that the phenotypic characteristics of depression in women are more complex. Significant heterogeneity in baseline depressive severity and longitudinal symptom progression reflects substantial individual differences, influenced by multiple psychological, social, and physiological factors. Moreover, the heterogeneity of depressive symptoms in women is more pronounced, highlighting the need for personalized intervention strategies. In contrast, male patients exhibit a slightly positive intercept-slope correlation, suggesting that those with higher initial depressive severity may experience faster symptom deterioration. This disease progression pattern may be linked to behavioral tendencies in men such as symptom concealment, delayed help-seeking, and emotional suppression—behaviors that could lead to accumulated psychological burdens and ultimately clinical worsening. Therefore, early detection and timely intervention are particularly crucial for male patients.

Ultimately, these research findings reveal sex-specific stratification characteristics in Parkinson’s disease (PD)-related depression, where neuroendocrine states—particularly the estrogen environment in female populations—significantly shape neuroanatomical and neurochemical responses triggered by neurodegenerative lesions. This hormonal foundation confers unique stress resilience in women, while men exhibit earlier structural atrophy, weakened functional connectivity, and reduced monoamine integrity in emotional regulation circuits. However, this study has several limitations: the current observation period was limited to four-year annual follow-ups, which may not fully track sex-specific trajectories of depressive symptoms and cognitive decline in PD patients; additionally, key environmental factors such as social support, socioeconomic status, and psychosocial stress were not considered. Future research should extend observation periods, track co-evolution patterns of depression and cognition through sex stratified tracking, and analyze longitudinal changes in levodopa equivalent daily dose (LEDD) and comorbidity burden. By integrating multimodal neuroimaging techniques (including PET imaging of serotonin transporter SERT and dopamine transporter DAT, as well as resting-state and task-based functional connectivity MRI) with continuous hormone monitoring (e.g., estradiol and follicle-stimulating hormone), we can further elucidate the complex interactions between endocrine states, neural circuits, and emotional outcomes. This comprehensive research approach will establish a solid scientific foundation for developing sex-specific predictive, preventive, and management strategies targeting PD-related depression.

## 5 Conclusion

This study, through sex-stratified analysis, revealed sex differences in the factors influencing depressive symptoms among patients with Parkinson’s disease. Anxiety levels (stai), activities of daily living (updrs2_score), and autonomic dysfunction (scopa_ur) were significant predictors of depression in both men and women; however, the magnitude of their effects differed by sex. Female patients exhibited stronger associations, as evidenced by higher regression coefficients for anxiety (mean_stai, standardized: *β* = 2.047 vs. 1.409 in males), anxiety fluctuation (stai_centered, standardized: *β* = 0.080 vs. 0.070), and scopa_ur (*β* = 0.084 vs. 0.063). Baseline disease duration (standardized) showed a marginally significant positive association with depressive symptoms in women (*β* = 0.203, *p* = 0.094) but no association in men (*β* = −0.006, *p* = 0.936). A significant protective effect of cognitive function (moca) against depression was observed exclusively in women (*β* = −0.074, *p* = 0.009), with no evidence of association in men (*β* = −0.015, *p* = 0.492).

Random-effects models further demonstrated sex heterogeneity in the trajectory of depression. Women exhibited substantially greater individual variability in baseline depression levels (intercept SD = 1.659, variance = 2.752) compared to men (intercept SD = 1.019, variance = 1.037), and greater heterogeneity in the rate of change over time (slope SD = 0.465, variance = 0.216 vs. 0.194, variance = 0.038 in men). Critically, women showed a strong negative intercept–slope correlation (*r* = −0.62), indicating that those with higher baseline GDS scores tended to have flatter or declining trajectories over time. In contrast, men showed near-zero correlation (*r* = 0.01), suggesting no systematic relationship between baseline severity and subsequent change.

## Data Availability

The original contributions presented in the study are included in the article/supplementary material, further inquiries can be directed to the corresponding author.

## Footnotes

1 https://www.ppmi-info.org/access-data-specimens/download-data
